# Yield Responses of Wheat to Mulching Practices in Dryland Farming on the Loess Plateau

**DOI:** 10.1371/journal.pone.0127402

**Published:** 2015-05-28

**Authors:** Li-fang Wang, Juan Chen, Zhou-ping Shangguan

**Affiliations:** State Key Laboratory of Soil Erosion and Dryland Farming on the Loess Plateau, Northwest A&F University, Yangling, Shaanxi, P.R. China; Chinese Academy of Sciences, CHINA

## Abstract

Improving farming practices of soil and water conservation has profound effects on the yield of wheat (*Triticum aestivum* L.) in dryland farming regions of the Loess Plateau in China. Mulching has proven to be an effective practice to increase crop yield, and possibly contribute to replenishing groundwater. This evaluation study collected and analyzed the data of 1849 observations published in 38 papers using meta-analysis to investigate effects of the mulching practices on wheat yield in terms of different rainfall and regions in comparison with conventional tillage. The main results of the study follow. The effects of the mulching practices were ranked in the order of RFM (ridge–furrow mulching) > MTMC (mulching with two materials combined) > MOM (mulching with other materials) > WSM (wheat straw mulching) > FM (flat mulching). The effects of the mulching practices at the different levels of rainfall during the wheat growing season were in the order: (< 150 mm) > (> 250 mm) > (150–250 mm). The effects of the mulching practices in the different regions were in the order of Henan > Shanxi > Shaanxi > Gansu. WSM, MTMC and FM performed better in improving wheat yield for rainfall of < 150, 150–250 and > 250 mm during the growing season, respectively. The wheat yield with FM, MTMC, MOM and MOM was higher than those with the other mulching practices in Shaanxi, Gansu, Henan and Shanxi. The wheat yield with RFM was 27.4% higher than that with FM, indicating that RFM was the most effective practice to improve wheat yield among all the practices. These findings have important implications for choosing appropriate crop field management to improve wheat yield.

## Introduction

In China, dryland farming is practiced on about one-third of the arable land. Water stress is the main factor limiting plant growth and crop yield in arid and semi-arid environments [1]. For farmland of the Loess Plateau, rainfall is the only reliable water source because of insufficient groundwater resources. However, annual rainfall on the Loess Plateau is in the range of 200–600 mm [2]. In some regions of the Loess Plateau, wheat (*Triticum aestivum* L.) yield is as low as 1500 kg ha^-1^ due to water deficits, which is one-third of the national average [3]. Thus, current crop production can be substantially increased, perhaps threefold, by optimizing soil water and nutrient management [4]. The challenges for increasing wheat yield in Northwest China are low water use efficiencies (WUEs) resulting from low and erratic rainfall, and removal of crop residues from farmlands as feed or cooking fuel [5,6]. With the human population in the Loess Plateau continuously increasing, it is crucial to improve crop yields in dryland farming by developing effective crop and water management practices to satisfy the needs of human survival and promote sustainable development of agriculture in the future.

In the Loess Plateau, winter wheat is sown in mid-September and harvested in early July of the following year. The winter wheat growing period does not coincide with the rainy season of June–September, so that 47% of its yield depends strongly on in-season rainfall [7]. Traditionally, Chinese farmers till soil after harvest by means of moldboard plow and fallow soil without mulching during the rainy season (July–September). Such practices cause considerable losses of soil moisture stored during the rainy season, lead to poor soil physical condition and negatively influence soil chemical properties [8]. Shangguan et al. [6] attributed low available soil water to high potential soil evaporation resulting from fallowing without mulching and over-tillage, and found that the rainfall storage efficiencies of fallow farmlands were in the range of only 35–40% under traditional tillage in the Loess Plateau, which further influences crop yield formation [9]. Plastic film mulching has proved an effective farming practice for improving soil water management, increasing soil moisture, promoting crop growth and increasing crop yield in the semi-arid region of the Loess Plateau [10–12]. Soil mulching with wheat straw, gravel or sand are regarded as effective ways of retaining more water in soil, decreasing soil evaporation and modifying microclimates and growing conditions of crops [13,14]. Crop yield differences are also related to different regional environments and crop varieties [15], and thus it is necessary to understand regional mulching differences so as to develop and improve mulching practices in different regions. It is important to quantitatively meta-analyze the limited information concerning the effects of different types of mulching on wheat yield.

Although numerous studies have been conducted to investigate effects of mulching practices on wheat yield, there have not been any integrated analyses of their relevant data. The present study collected data of 1849 observations carried out in 38 individual studies and meta-analyzed them to identify the general patterns of different mulching practices affecting wheat yield at different levels of rainfall in different regions. The objectives of the study were (1) to quantify responses of wheat yield to five different mulching practices; and (2) to compare the yield differences between the conventional farming and mulching practices at different levels of rainfall in regions.

## Materials and Methods

### Data search and collection

Relevant literature on wheat yields versus mulching practices, rainfall and regions was searched in internet databases in the Chinese Academy of Sciences (http://www.isiknowledge.com/ and http://www.cnki.net/). A Preferred Reporting Items for Systematic Reviews and Meta-Analyses (PRISMA) checklist was applied (S1 Table). When the data in question were expressed in the forms of figures or charts, they were transformed into numerical values depending on their digital versions using Get-Data Graph Digitizer (ver. 2.24, Russian Federation). To avoid any distortions caused by printing, the data chosen to be transformed satisfied the following criteria: (i) field experimental studies involved mulching treatments and controls as well as three variables in question (mulching, regions and rainfall); (ii) wheat was not irrigated during the whole wheat growing season; and (iii) for multifactorial studies, only data of control and mulching treatments were cited and the interactions between treatments were excluded. Additionally, the means, standard deviations (or standard errors) and sample sizes of the variables concerned were directly available or could be calculated depending on the data of the studies involved.

**Fig 1:**
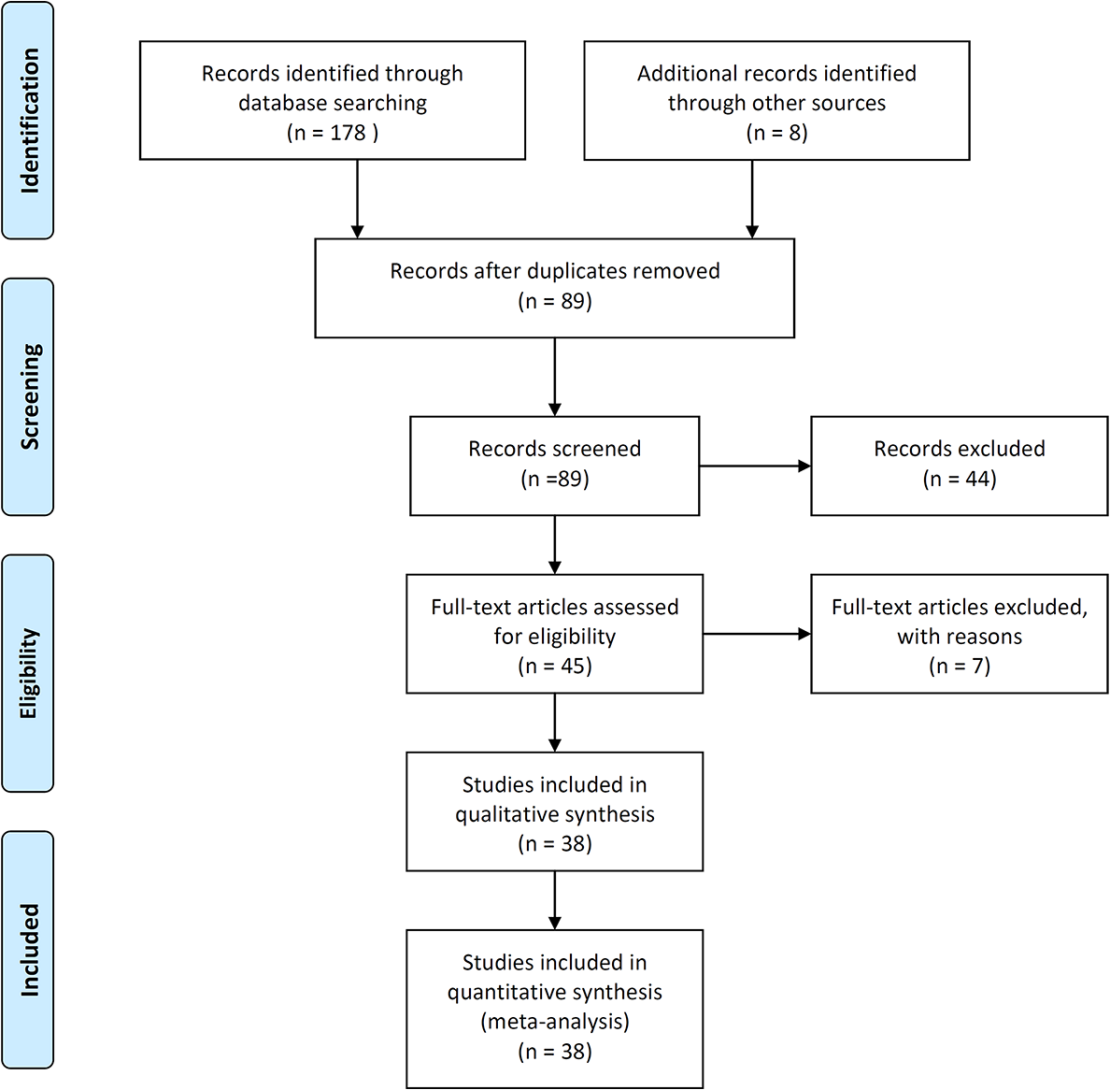
Flow diagram reporting the number of records identified, excluded, and added during the screening process.

The search was performed in June 2014, yielding a total of 178 studies. We obtained additional 8 studies from colleagues. After removal of duplicates, 89 studies remained. Study selection was a three-stage process. First, 57 studies with relevant titles were selected. Second, selection was made based on abstracts, after which 45 studies remained. Full paper content was assessed in the third stage, leaving 38 studies (including 1849 observations at 18 sites) for the meta-analysis after screening some data no published (Fig 1, see S2 and S3 Tables for database and references).

**Fig 2:**
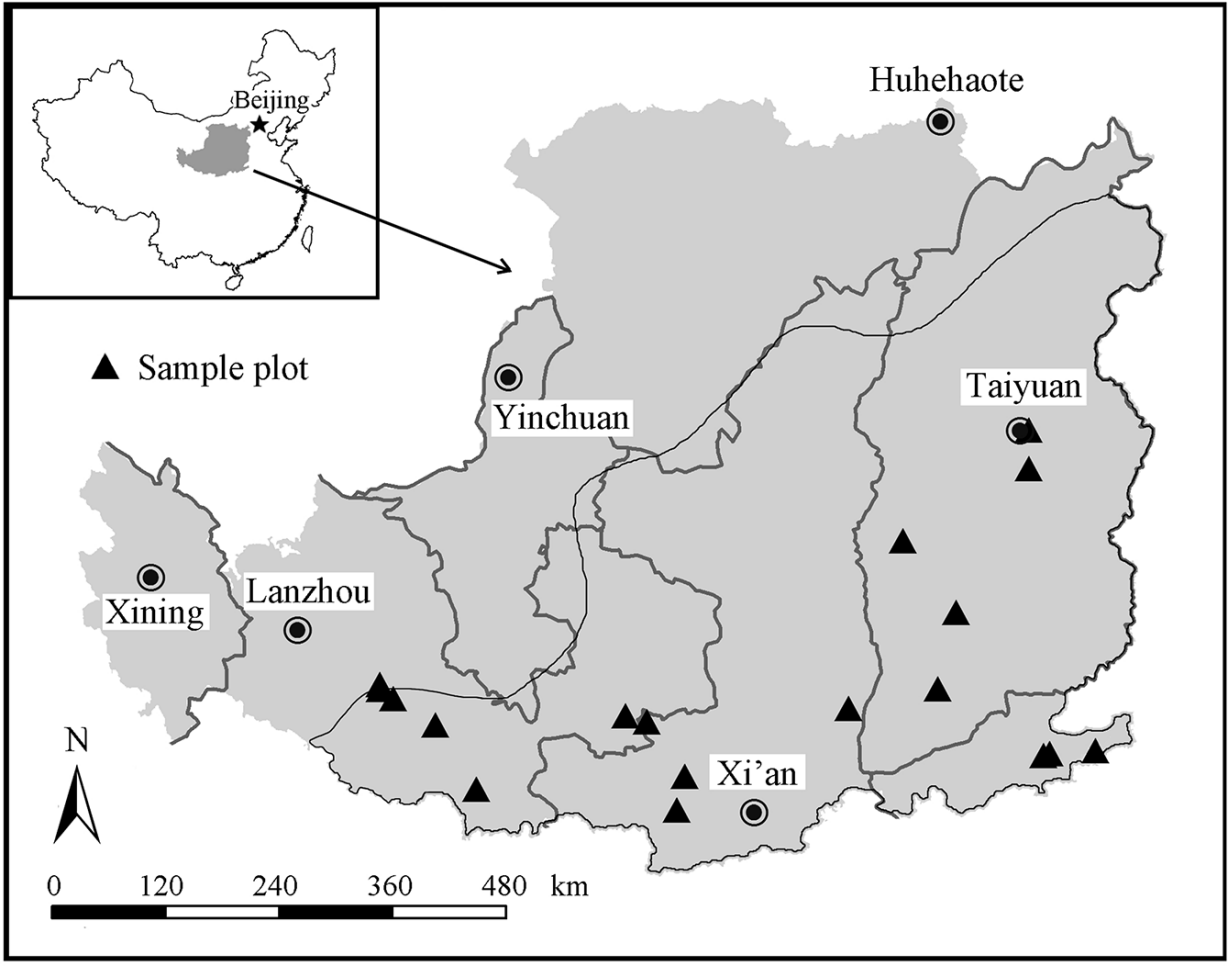
Map of the Loess Plateau with a map of China enclosed on its upper left. The locations of the field experiments of wheat involved in the peer–reviewed literatures in the meta-analysis are shown on the map. The area below the black southeastern line is the typical winter wheat planting regions in the Loess Plateau.

The wheat yields (kg ha^-1^) were aligned with four different regions of the Loess Plateau: Shaanxi, Gansu, Henan and Shanxi (Fig 2). The field management practices were grouped into five types: CT, FM, RFM, WSM, MOM and MTMC (see Table 1 for details). Three rainfall levels were used during the period of crop growth involved (< 150, 150–250 and > 250 mm); and if no exact rainfall data were available, they were estimated using relevant reported monthly rainfall. Average monthly climatic characteristics data of four sites (Table 2) were taken from China Data Sharing Infrastructure of Earth System Science of Loess Plateau (http://loess.data.ac.cn/). The soil types of the four regions were all considered suitable for crop production, and the basic soil characteristics were presented in Table 3.

**Table 1:** A brief description of the different mulching and conventional tillage practices in wheat field experiments.

Mulching practice and tillage	Brief description
Conventional tillage (CT)	Plowing and fallowing without mulching until the next sowing time, a farming practice most widelypracticed by local farmers (no mulching)

Flat mulching (FM)	Keep soil surface flat and mulching it with plastic film of 7 or 8 μm thickness without color
Ridge–furrow mulching (RFM)	Preparing soil surface into ridges and furrows and mulching the ridges with plastic film to concentraterainfall into the furrows; the ridge height was 5–40 cm and ridge bredth was 20–60 cm

Wheat straw mulching (WSM)	Evenly covering soil surface with wheat straw, chopped or not chopped at coverage rate of 1–9 t ha^-1^over the location

Mulching with other materials(MOM)	Mulching with liquid film (450 kg ha^-1^), water-permeability plastic film or sand (1–2 cm)

Mulching with two materials combined (MTMC)	Plastic film + wheat straw; liquid film + straw; and plastic film + liquid film


**Table 2:** Average monthly rainfall, pan evaporation and maximum and minimum temperature at the experimental sites of Shaanxi, Gansu, Henan and Shanxi.

	January	February	March	April	May	June	July	August	September	October	November	December
Shaanxi												
*T*_max_ (°C)	2.6	5.8	12.2	19.0	24.4	29.7	30.1	29.0	22.9	17.7	10.3	4.0
*T*_min_ (°C)	−8.8	−5.6	0.5	6.4	11.2	17.5	19.2	18.3	12.6	6.6	−0.7	−6.7
Rainfall (mm)	4.5	8.2	19.9	42.4	51.7	49.7	110.6	97.3	94.7	50.2	21.5	4.4
Pan evaporation (mm)	50.6	69.6	111.0	189.5	228.1	268.2	220.2	187.2	134.9	98.9	63.7	48.6
Gansu												
*T*_max_ (°C)	1.1	3.8	10.2	16.9	21.2	25.1	26.5	25.4	19.7	14.7	7.7	2.4
*T*_min_ (°C)	−10.1	−7.0	−0.7	4.3	8.6	11.7	14.7	14.1	9.7	4.3	−2.2	−8.1
Rainfall (mm)	3.0	4.8	12.9	31.5	49.0	50.3	96.7	99.1	79.2	37.9	12.0	2.3
Pan evaporation (mm)	0.5	41.7	57.0	117.6	180.0	210.1	222.6	208.7	186.1	115.0	89.5	55.3
Henan												
*T*_max_ (°C)	5.8	8.3	14.3	21.2	27.0	32.3	31.6	30.4	26.4	21.4	14.1	7.5
*T*_min_ (°C)	−4.4	−2.2	2.5	8.9	13.8	19.4	22.3	21.4	15.8	10.0	3.2	−2.2
Rainfall (mm)	7.2	11.7	23.9	50.7	45.4	65.1	146.9	99.1	84.4	47.4	27.6	7.7
Pan evaporation (mm)	70.1	81.1	135.9	175.7	232.9	289.6	204.3	177.8	139.7	128.7	93.0	76.5
Shanxi												
*T*_max_ (°C)	0.2	3.6	10.4	18.0	24.4	28.6	28.8	27.4	22.5	17.0	8.7	1.8
*T*_min_ (°C)	−11.1	–8.6	−1.9	4.1	9.8	14.6	17.8	16.4	10.3	4.1	−2.9	−10.1
Rainfall (mm)	3.3	5.7	11.6	23.8	29.1	55.1	124.2	108.8	67.1	30.7	13.1	3.5
Pan evaporation (mm)	46.8	65.3	128.5	212.2	292.6	299.6	224.9	190.3	144.0	120.3	70.8	46.7

**Table 3:** Basic soil characteristics (0–20 cm) at the experimental sites. N: nitrogen; P: phosphorus; K: potassium.

Site	Soil type	Organic matter	Bulk density	Total N	Available N	Available P	Available K
		(g kg^-1^)	(g cm^-3^)	(g kg^-1^)	(mg kg^-1^)	(mg kg^-1^)	(mg kg^-1^)
**Shaanxi**	Eum-orthic Anthrosol, silt loam	12.8	1.3	0.8	57.0	11.49	141.2
**Gansu**	Loess Orthic Entisol	8.8	1.2	1.0	51.3	8.6	121.9
**Henan**	Light loam	15.8	1.5	0.8	62.7	10.4	166.0
**Shanxi**	Cinnamon loess soil, sandy loam	19.4	1.3	1.0	43.3	35.6	216.1

### Data processing

The data were analyzed using the meta-analysis methods described by Hedges et al. [16]. The effects of mulching practices, rainfall and regions for each individual observation of yield were estimated by the SMD (standardized mean difference, g):

$$g\;=\;\frac{({\bar{X}}_{E}-{\bar{X}}_{C})}{S_{within}}\;J$$

$$S_{within}\;=\;\sqrt{\frac{(N_{E}-1){\left(S_{E}\right)}^{2}\;+\;(N_{C}-1){\left(S_{C}\right)}^{2}}{N_{E}\;+\;N_{C}\;-2}}$$

$$J\;=\;1-\;\frac{3}{4(N_{C}\;+\;N_{E}\;-2)-1}$$

$$V_{g}\;=\;(\frac{N_{C}\;+\;N_{E}}{N_{C}N_{E}}\;+\;\frac{d^{2}}{2(N_{C}\;+\;N_{E})})J^{2}$$

where ${\bar{X}}_{E}$ and ${\bar{X}}_{C}$ are the means of the treatment and control groups, respectively; *N*_*E*_ and *N*_*C*_ are the sample sizes for the treatment and control groups, respectively; *Vg* is the variance of independent research; and *S*_*E*_ and *S*_*C*_ are the standard deviations for all comparisons in the treatment and control groups, respectively. Several studies did not report standard deviations; in these cases, we calculated the average coefficient of variation (CV) within each data set and then approximated the missing standard deviations by multiplying the reported means by the average CVs.

In the study, the indices concerned were continuous variables and their SMDs were calculated by meta-analysis to indicate the effects of mulching on wheat yield. The yield differences of wheat among the different mulching practices, rainfall during the growing season, and regions were calculated by the fixed or random-effects models using the Review Manager program (RevMan version 5.2, 2012; Cochrane Collaboration). In this study, the inverse variances and SMDs were adopted as the statistical methods and effects for the meta-analysis, respectively. Random-effects models were adopted where there were moderate-to-high heterogeneities (indicated by *X* ^2^ > 50% and P < 0.05) [17]. The mean differences of the different experiments with the conventional tillage (CT) and mulching practices were weighted according to their sample sizes and SD, as determined by the RevMan program and accordingly the confidence intervals (CI) on their weighted effects were generated. If the 95% CI values of the effect size for a variable did not reach zero, the effect of mulching on the variable was considered as differing significantly between two treatments.

## Results

**Fig 3:**
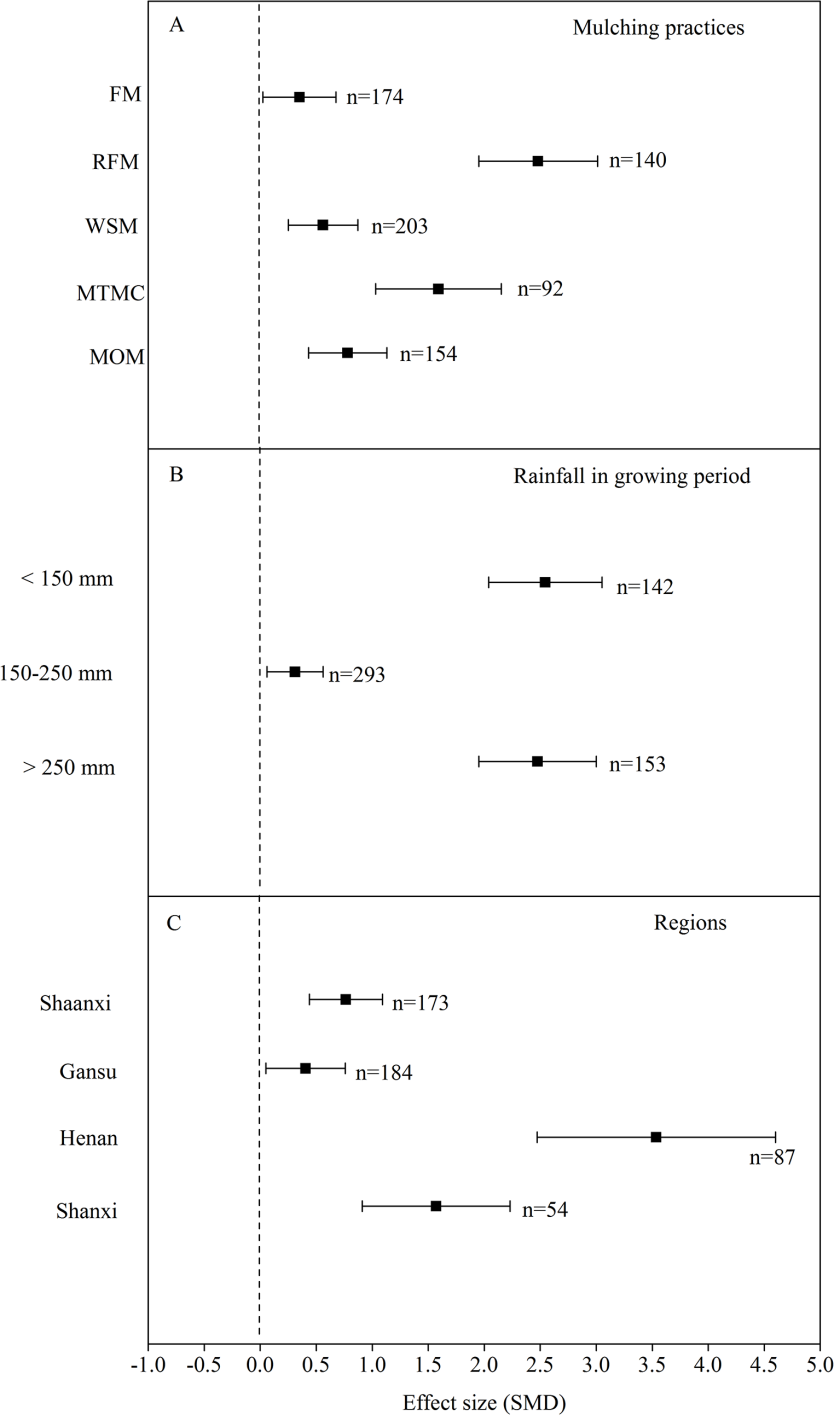
Relative yields of wheat for the mulching practices in the Loess Plateau. (A) The five mulching practices, (B) three rainfalls during the wheat growing period and (C) the four regions. The error bars stand for the 95% CI, and the values close to the bars are the corresponding number of observations. FM: flat mulching; RFM: ridge–furrow mulching; WSM: wheat straw mulching; MTMC: mulching with two materials combined; MOM: mulching with other materials.

**Fig 4:**
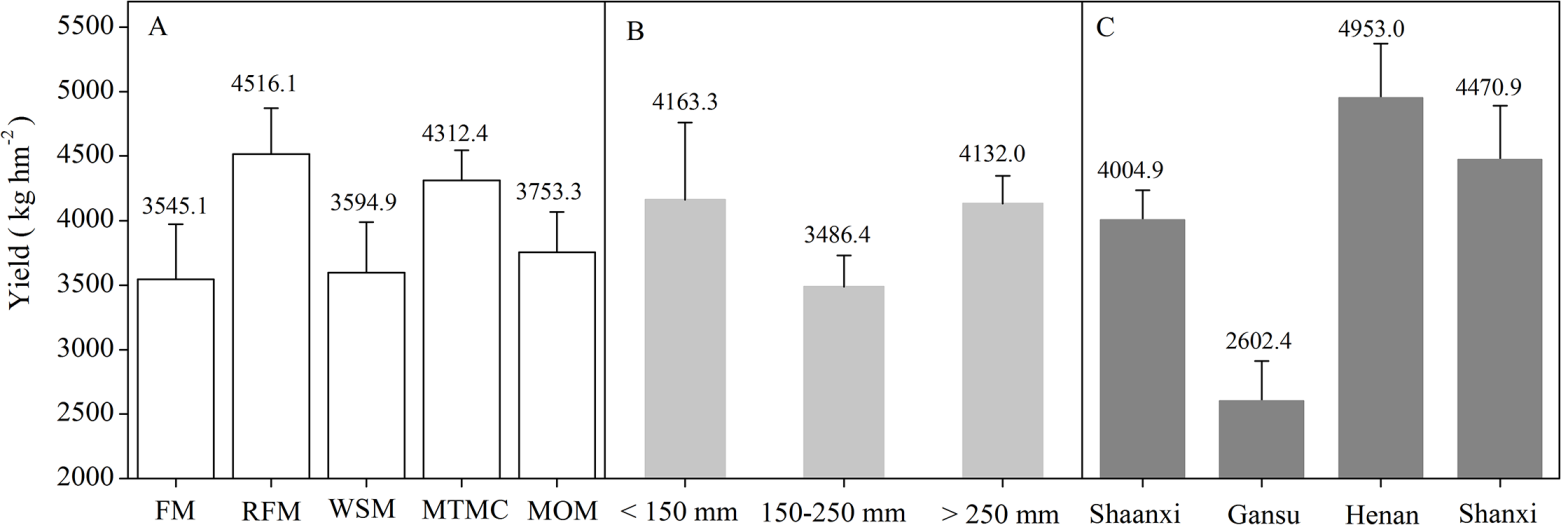
Yields of wheat versus mulching practices. (A) The five mulching practices, (B) the three rainfalls during the wheat growing period and (C) the four regions. FM: flat mulching; RFM: ridge–furrow mulching; WSM: wheat straw mulching; MTMC: mulching with two materials combined; MOM: mulching with other materials.

The meta-analysis of the data in the 18 experiment sites during 1979–2014 indicated that the overall effects of the mulching practices significantly increased the yield of wheat (Fig 3, Table 4). The mean effects of FM, RFM, WSM, MTMC and MOM were 0.34 (95% CI: 0.02–0.67), 2.48 (95% CI: 1.95–3.01), 0.56 (95% CI: 0.25–0.87), 1.59 (95% CI: 1.03–2.15) and 0.78 (95% CI: 0.43–1.13), respectively (Fig 3A). Compared with CT, the effects of the mulching practices had CI > 0, suggesting that the mulching practices had significant positive effects on wheat yield. The effects of the different mulching practices were ranked in the order of RFM > MTMC > MOM > WSM > FM. Wheat yield for RFM was 27.4% higher than for FM (Fig 4A), and 22% higher than for CT.

**Table 4:** Meta-analysis results on the wheat yields for five mulching practices, three rainfalls and four regions using the fixed or random-effects models.

Item	Categorical		Heterogeneity			
	variable	K	Qwi	df	P-value of	I^2^
					chi-square test	(%)
Mulching	FM	173	18.83*	13	0.13	31
practice	RFM	139	14.60**	11	0.2	25
	WSM	202	41.89**	13	< 0.0001	69
	MOM	91	12.95**	7	0.07	46
	MTMC	153	13.61**	8	0.09	41
Rainfall	< 150 mm	141	16.90**	9	0.05	47
	150–250 mm	292	34.46*	23	0.06	33
	> 250 mm	152	19.64**	15	0.19	24
Region	Shaanxi	172	17.63**	12	0.13	32
	Gansu	153	20.57*	13	0.08	37
	Henan	86	27.39**	6	0.0001	78
	Shanxi	53	1.00**	3	0.8	0

Notes: FM: flat mulching; RFM: ridge–furrow mulching; WSM: wheat straw mulching; MTMC: mulching with two materials combined; MOM: mulching with other materials. K: the number of observations involved in each of the analysis levels including conventional tillage (CT). Several individual studies were excluded because their heterogeneities could not be eliminated.

* P < 0.05;

** P < 0.01.

The mean effect size of the mulching practices on wheat yield at rainfalls of < 150 mm, 150–250 mm and > 250 mm during the growing season were 2.55 (95% CI: 2.04, 3.05), 0.31 (95% CI: 0.06, 0.56) and 2.48 (95% CI: 1.95, 3.00), respectively, and the 95% CI did not cover zero (Fig 3B), indicating that the five mulching practices had significant positive effects on wheat yield for the different levels of rainfall. The effects at the different rainfalls during the growing season were ranked in the following order: (< 150 mm) > (> 250 mm) > (150–250 mm). Across all mulching practices, the wheat yield at rainfall of < 150 mm was 19.4% higher than for rainfall of 150–250 mm during the growing season (Fig 4B), and the wheat yield at rainfall of < 150 mm across all mulching practices was 22% higher than for CT.

The yield of wheat in different regions showed different responses to the mulching practices (Fig 3C). There were less < 10 publications involving mulching practices and wheat yield in Henan and Shanxi; in Henan, wheat yield was most affected by mulching practices (mean effect size = 3.71; 95% CI: 2.81–4.60), based on the limited number of samples collected. The effects in the different regions were ranked in the order of Henan (with mean effect size at 3.71 and 95% CI in the range of 2.81–4.60) > Shanxi (mean effect size 1.57, 95% CI of 0.91–2.23) > Shaanxi (mean effect size 0.77, 95% CI of 0.44–1.09) > Gansu (mean effect size 0.40, 95% CI of 0.05–0.76). For all mulching practices, the wheat yield in Henan was nearly twice that in Gansu (Fig 4C); and in Henan, the yields of wheat across all mulching practices increased by 10% compared with those of CT.

**Fig 5:**
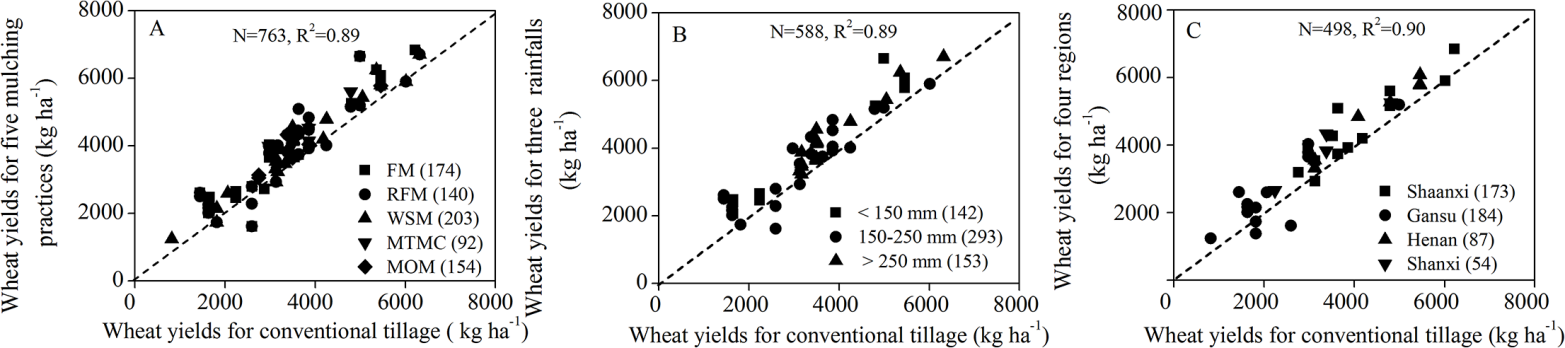
Relationships of wheat yields between mulching and conventional practices. (A) The five mulching practices, (B) the three rainfalls during the wheat growing period and (C) the four regions. In the figure, each black point represents one individual comparison result between the yields of wheat for the mulching practices and the control. The points lying on the 1:1 line indicate that the yield of wheat responded similarly to the mulching practices and the control, whereas the points distributed above or below the line indicate a positive or negative effect of the mulching practices on the yield of wheat, respectively. FM: flat mulching; RFM: ridge–furrow mulching; WSM: wheat straw mulching; MTMC: mulching with two materials combined; MOM: mulching with other materials.

Of the 1849 observations analyzed in the meta-analysis, 763 involved the five mulching practices, 588 involved the three levels of rainfalls during the growing period and 498 involved the four regions, and all showed significant promoting effects. The wheat yields of the different mulching practices obviously increased compared with controls (Fig 5A). The slopes and coefficients of the correlations between the wheat yields and the mulching practices, the rainfall during the growing season and the regions were similar, which supported the view that mulching and rainfall were equally important in these regions of the Loess Plateau (Fig 5).

**Fig 6:**
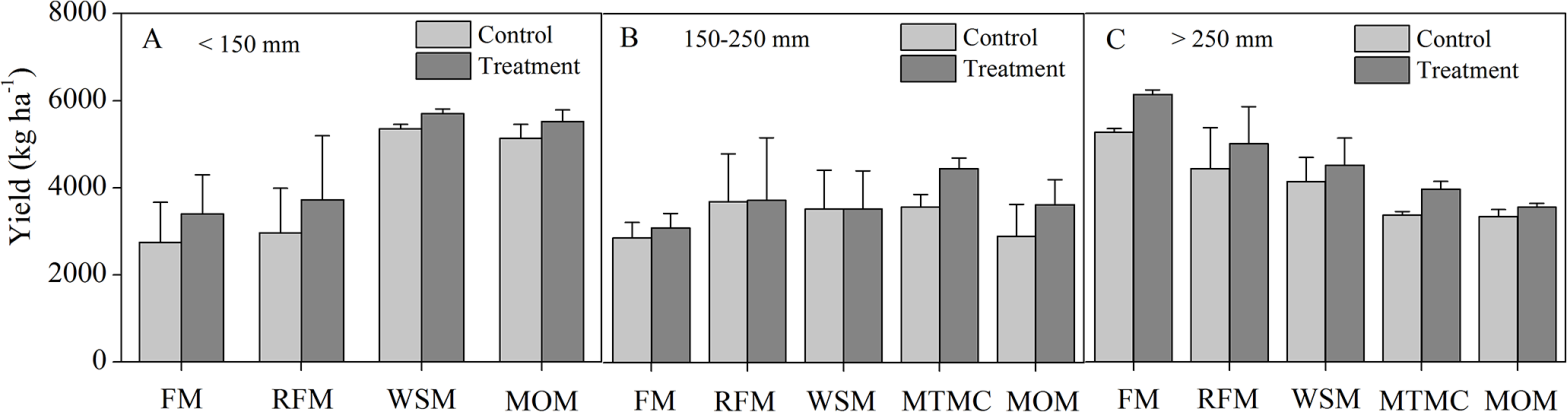
Wheat yields for the different mulching and conventional practices at different levels of rainfall during the growing period. (A) < 150 mm, (B) 150–250 mm and (C) > 250 mm. FM: flat mulching; RFM: ridge–furrow mulching; WSM: wheat straw mulching; MTMC: mulching with two materials combined; MOM: mulching with other materials.

Wheat yields of the five mulching practices at the three levels of rainfall are shown in Fig 6. At the rainfall of < 150 mm during the growing season, wheat yield benefited greatly from WSM and MOM among the different mulching practices (Fig 6A). WSM performed better than the other three measures at the rainfall of < 150 mm. Wheat yield of WSM was 3.37, 53.27 and 67.67% higher than for MOM, RFM and FM, respectively, and wheat yield of RFM was 761.84 kg ha^-1^ higher than controls. MTMC performed better than the other four mulching practices at the rainfall of 150–250 mm (Fig 6B). Wheat yield of MTMC was 44.06, 19.63, 26.17 and 22.77% higher than of FM, RFM, WSM and MOM, respectively. Moreover, the wheat yield of MTMC was 885.67 kg ha^-1^ higher than for controls. FM performed better than RFM, WSM, MTMC and MOM at the rainfall of > 250 mm (Fig 6C), and wheat yield of FM was correspondingly 22.47, 35.92, 54.78 and 72.76% higher; and the yield of FM was 861.89 kg ha^-1^ higher than of controls.

**Fig 7:**
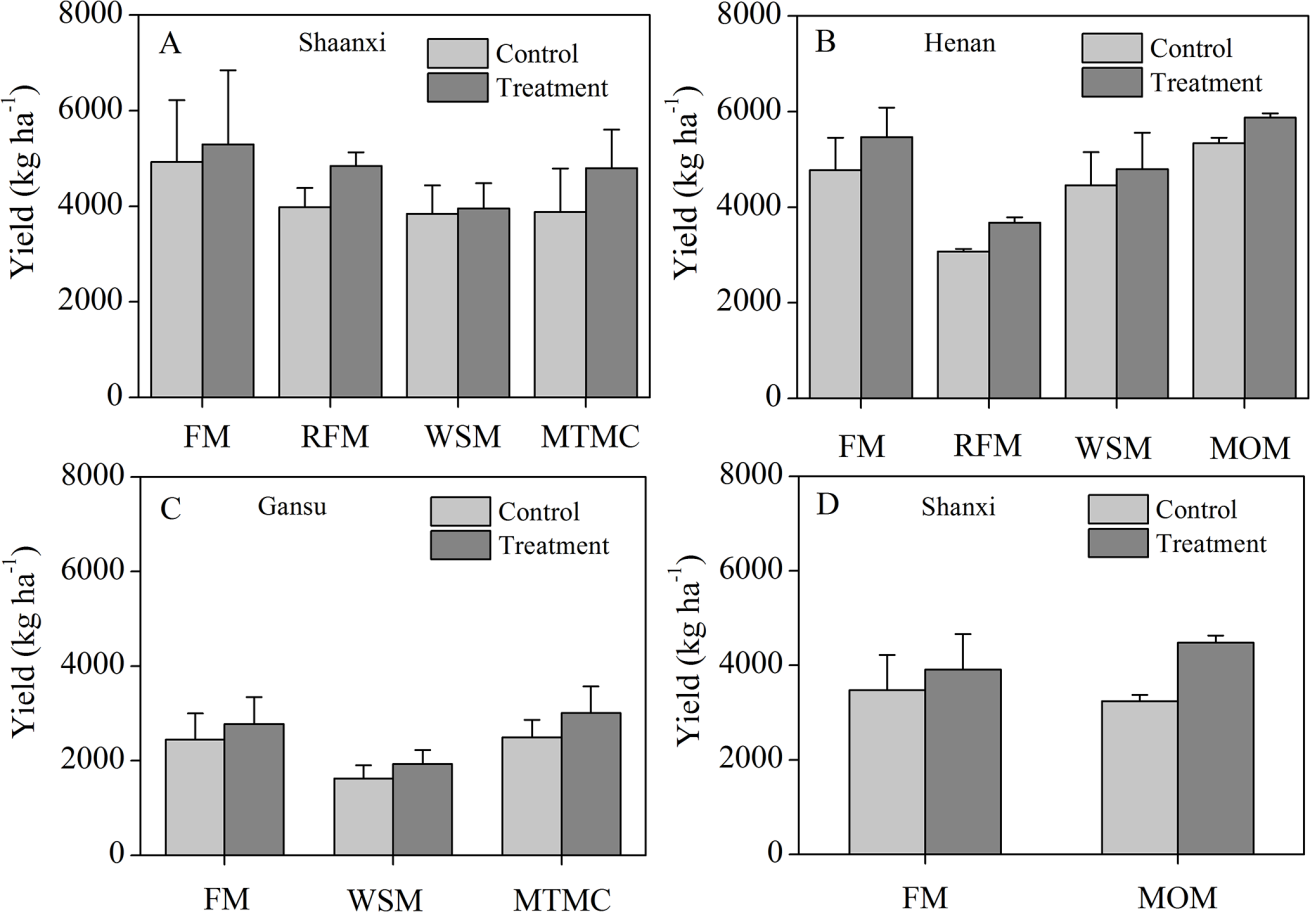
Wheat yields for the different mulching and conventional practices in different regions. (A) Shaanxi, (B) Gansu, (C) Henan and (D) Shanxi. FM: flat mulching; RFM: ridge–furrow mulching; WSM: wheat straw mulching; MTMC: mulching with two materials combined; MOM: mulching with other materials.

The limited available data showed that the different mulching practices were extensively adopted in the four regions (Fig 7). FM, RFM, WSM and MTMC were commonly adopted in Shaanxi; FM, WSM and MTMC in Gansu; FM, RFM, WSM and MOM in Henan; and FM and MOM in Shanxi. The yields were higher with FM than with the other mulching practices in Shaanxi, Henan, Gansu and Shanxi. The average wheat yields were 4720, 2573, 4953 and 4196kg ha^-1^ in Shaanxi, Gansu, Henan and Shanxi, respectively.

## Discussion

The relationship between soil water storage and wheat yield indicated the importance of practices in harvesting and retaining seasonal rainfall (Fig 5C). Greater water storage with mulching means more available water for wheat growth so that the stomata of wheat plants can open more freely and generate higher WUE relative to that for conventional practice [18]. Plastic film mulching can promote earlier seed germination and earlier spike differentiation [19,20]; improve root weight, and extend root distribution [21]; increase tiller number, growing period, spikelet and grain numbers per spike, and grain filling period, which is beneficial for wheat to allocate and transport assimilates stored in vegetative organs into reproductive organs [22]. Owing to its many advantageous effects, mulching improves both crop yield and quality [23] (Figs 2A and 5A). Therefore, mulching played an important role in improving plant growth, especially on farmland in semi–arid regions of the Loess Plateau where rainfall is the most important water source for wheat or other crops.

### Yield responses to mulching practices

Some studies have shown that prevailing mulching practices commonly increase the seed and fruit yields of a wide range of agricultural species compared with conventional practices [3,24], and this is important for food security and economic development in the dryland farming practiced in Northwest China. The meta-analysis of the peer-reviewed literature indicated that mulching practices had positive effects on wheat yield (Fig 3A). Without mulching, the lower yield of wheat for CT could be attributed to compact topsoil and exacerbated surface runoff and erosion [18,25,26]. RFM increased the wheat yield by up to 22% compared with CT and its effect was the greatest of the different mulching practices. Furthermore, in 18 experimental sites of the Loess Plateau, the MTMC, MOM and WSM exerted greater influence on wheat yield, and FM showed a weaker effect (Fig 3A). The wheat yield for RFM was 27.4% higher than for FM (Fig 4A). Similarly, RFM effectively improved yield of winter wheat in the Wei Bei highlands of northwest China, and demonstrated that it was a suitable farming practice for winter wheat in this region [27].

An extensive literature review on the effects of mulching practices on crop yield showed that mulching practices tended to increase wheat yield compared to CT (Fig 3). This was likely because film mulching prevented soil evaporation thereby causing higher soil temperature, which played a role in soil water re-distribution, and the effects of film mulching on soil temperature appear to be very important to improving water–temperature conditions and crop yield formation [28]. RFM reduces soil water evaporation by blocking water vapor exchange at the soil–atmosphere interface [29], and increases water harvested during small rainfall events (< 5 mm) [30]. This would improve water availability to plants compared with flat-farming practices [28], so that both the seed number per spike and the 1000-seed weight of wheat for RFM reached their maximum and as a result the wheat yields for RFM were obviously higher than for the other treatments (Fig 3A). In the dryland farming regions of the Loess Plateau, MTMC reduced soil evaporation, and promoted rainfall infiltration into soil, which improved soil water retention. MTMC is recommended as an efficient means for increasing crop yield and maintaining or improving soil fertility in semi-arid regions [28,31]. Higher soil moisture and increased yield of wheat resulting from straw mulching were previously reported in India and Bangladesh [32,33]. With the global drive to increase soil carbon by retaining crop residues in soil, it is important to further research the physiological consequences of retaining water by mulching. The effects of the different mulching practices were ranked in the order of RFM > MTMC > MOM > WSM > FM (Fig 3A), which was not consistent with Chen’s result [34] that the effect of straw mulching on soil water storage was more evident than that of ridge-formed tillage—these discrepancies of different mulching practices were probably due to differences in soil, climate conditions and crop planting and in particular test duration. Additionally, Gao et al. [35] showed that the instability of production increases due to straw mulching was probably related to many factors, such as soil moisture, fertility and temperature. Therefore, it is worth further investigating the responses of various crop varieties to WSM where WSM is suitable.

### Yield responses to mulching practices at different levels of rainfall during the growing season

Highly effective exploitation of rainfall and water balance is crucial for the sustainability of agro-ecosystems in semi-arid regions, where crop production generally depends on rainfall and thus it is crucial to conserve soil moisture for grain production [36]. The relationship between soil water storage and crop yield indicates the importance of practices to harvest seasonal rainfall, and accumulated rainfall resulting from mulching is beneficial to replenish groundwater during the crop growing season [37]. In Northern China, where the rotation of winter wheat–summer maize is practiced, seasonal rainfall and its distribution play an important role at all growth and development stages of maize, especially in the month before its pollination [38]. The present study showed that the effects of the different levels of rainfall during the growing period were ranked in the order of (< 150 mm) > (> 250 mm) > (150–250 mm) (Fig 3B), which were not consistent with Zhou’s result [28] that mulching exhibited great potential in crop production with limited rainfall. This difference shown by (> 250 mm) > (150–250 mm) was probably because the effects of FM and WSM at the rainfall of 150–250 mm were less than for MTMC at rainfall of > 250 mm (Fig 3A); Due to the soil in Loess Plateau with characteristics of deep, porous and homogenous, the rainfall can penetrate to depths below the depth of rooting and contributed to deep drainage to improve soil water content and soil temperature under mulching practice when the rain rich [28, 39]; and the mulching system may affect the properties of soil due to increased crop straw and root residues returned to the soil which varies with rainfall. We consider that more in-depth research is needed to further study the mechanisms influencing the relationships among mulching materials, rainfall and soil characteristics.

This study also showed that the yield of WSM was higher than of FM, RFM and MOM at the rainfall of < 150 mm (Fig 6A); yield of MTMC was higher than of FM, RFM, WSM and MOM at 150–250 mm (Fig 6B); and yield of FM was higher than of RFM, WSM, MTMC and MOM at > 250 mm (Fig 6C). These results showed that the yield performance differences among the different mulching practices probably resulted from different soil water retention capacities of the different mulching practices.

### Yield responses to mulching practices for different regions

Of the mulching practices, the effects in the different regions were ranked in the order of Henan > Shanxi > Shaanxi > Gansu. The wheat yield in Henan was nearly twice that in Gansu (Fig 4C), and in Henan the mulching practices increased wheat yield by 10% compared with CT. The annual rainfall was higher in Henan than in the other regions of the Loess Plateau (S2 Table). Because there were very close relationships between soil moisture accumulation in the fallow period and wheat yield, the differences in the effects among the experiment sites were related to the rainfall or rain intensities in these regions.

### Uncertainty in meta-analysis

The representative data of the study, screened from literature obtained in long–term experiment sites, involved the main types of mulching practices used in the Loess Plateau. Differences in researcher experience and preference, experimental conditions and research methods to obtain similar types of data make it hard to eliminate inconsistencies among qualities of different types of reported data. This introduced uncertainties into the meta-analysis of wheat yield. In addition, the study could not exclude that some seasonal variations were caused by slight spatial variations in soil characteristics. The effects of the mulching practices on soil water storage and crop yield depend on soil type, climate, environmental conditions, fertilization and land type. The effect size proportions of the different mulching practices on wheat yield vary depending on factors affecting ground cover percentage (e.g. previous crops and crop varieties), field management (e.g. fertilization strategy and sowing time), climate and soil hydraulic properties [40]. At the same time, the dynamic yield changes of wheat yield arise from many interacting factors. Although the effect size and CI of the yield change of wheat can be quantified by meta-analysis to some extent, the underlying sources of meta-analysis uncertainties require in-depth study in the future.

## Conclusions

Evaluating the feasibility of mulching practices offers a wide range of solutions to guarantee both food security and system sustainability in the Loess Plateau, which suffers from water shortages. Using results of past research, the present study concluded the following. (1) The effects of the mulching practices on wheat yield were ranked in the order of RFM > MTMC> MOM > WSM > FM. The effects of the different levels of rainfall on wheat yield during the growing season were ranked in the order of (< 150 mm) > (> 250 mm) > (150–250 mm). The effects on wheat yield in the different regions were ranked in the order of Henan > Shanxi > Shaanxi > Gansu. (2) Correlation coefficients between the wheat yields and mulching practices, rainfall and regions were high and similar. (3) WSM performed better than FM, RFM and MOM at rainfall of < 150 mm during the growing season; MTMC performed better than FM, RFM, WSM and MOM at 150–250 mm; and FM performed better than RFM, WSM, MTMC and MOM at > 250 mm. More field research is needed to develop best performing mulching practices under different site conditions and so to provide guidelines for farmers to better arrange field management practices for wheat production.

## Supporting Information

S1 Table PRISMA (Preferred Reporting Items for Systematic Reviews and Meta-Analyses) checklist. (DOC)

S2 Table Database used in the meta-analysis, which plots the effect of mulching practices versus conventional tillage on wheat yield in the Loess Plateau, China. FM: flat mulching; RFM: ridge–furrow mulching; WSM: wheat straw mulching; MTMC: mulching with two materials combined; MOM: mulching with other materials.
(DOCX)

S3 Table Full references for the 38 studies included in the meta-analysis. (DOCX)
