# Supplementary material for: Yield Responses of Wheat to Mulching Practices in Dryland Farming on the Loess Plateau
Source: PLoS One. 2015 May 28;10(5):e0127402. doi: 10.1371/journal.pone.0127402 (PMC4447293; doi:10.1371/journal.pone.0127402)
Supplement: S2 Table — FM: flat mulching; RFM: ridge–furrow mulching; WSM: wheat straw mulching; MTMC: mulching with two materials combined; MOM: mulching with other materials. (DOCX) [file pone.0127402.s002.docx]

**S2 Table. Database used in the meta-analysis, which plots the effect of mulching practices versus conventional tillage on wheat yield in the Loess Plateau, China.** FM: flat mulching; RFM: ridge–furrow mulching; WSM: wheat straw mulching; MTMC: mulching with two materials combined; MOM: mulching with other materials.

| **Location** | **Latitude** | **Longitude** | **Annual mean**  **prec. (mm)** | **Annual growth period prec. (mm)** | **Practices** | **Wheat type** | **Experiment years** | **Reference** |
| --- | --- | --- | --- | --- | --- | --- | --- | --- |
| 1.Yangling, Shaanxi | 34°20' | 108°04' | 550 | 188. 01 | CT, FM,  RFM | Winter | 2010-2011 | Zhang et al. (2012a) |
| 2. Heyang, Shaanxi | 35°14' | 110°09' | 582 | 248.5 | CT, WSM | Winter | 2007-2009 | Liu et al. (2010) |
| 3. Heyang, Shaanxi | 35°14' | 110°09' | 582 | 184 | CT, WSM | Winter | 2001-2003 | Zhang et al. (2005) |
| 4. Heyang, Shaanxi | 35°15' | 110°10' | 582 | 249.47 | CT, RFM,  MTMC, MOM | Winter | 2007-2010 | Li et al. (2011) |
| 5.Yangling, Shaanxi | 34°20' | 108°04' | 550 | 120.9 | CT, RFM | Winter | 2000-2001 | Wang et al. (2004) |
| 6.Luoyang, Henan | 34°37' | 112°24' | 614 | 104.5 | CT, WSM | Winter | 2006-2007 | Wu et al. (2008) |
| 7.Changwu, Shaanxi | 35°12' | 107°45' | 584 | 186.5 | CT, MTMC | Winter | 1998-2003 | Dang et al. (2008) |
| 8.Heyang, Shaanxi | 35°14' | 110°09' | 571.9 | 254.65 | CT, RFM | Winter | 2007-2009 | Bai et al. (2010) |
| 9.Heyang, Shaanxi | 35°14' | 110°09' | 571.9 | 240.35 | CT, RFM,  MTMC | Winter | 2007-2009 | Liu et al. (2010) |
| 10.Heyang, Shaanxi | 35°15' | 110°10' | 534.6 | 248.5 | CT, WSM | Winter | 2007-2010 | Ma et al. (2010) |
| 11.Wenxi, Shanxi | 35°21' | 111°13' | 430 | 133.2 | CT, FM,  MOM | Winter | 2010-2011 | Wen et al. (2013) |
| 12.Changwu, Shaanxi | 35°12' | 107°45' | 578.5 | 255 | CT, FM | Winter | 2011-2012 | Yang et al. (2013) |
| 13.Qianxian, Shaanxi | 34°39' | 108°10' | 584.2 | 226 | CT, RFM,  MTMC | Winter | 2000-2001 | Liao et al. (2003) |
| 14.Heyang, Shaanxi | 35°14' | 110°09' | 571.9 | — | CT, FM,  WSM, MTMC | Winter | 2002-2004 | Fang et al. (2006) |
| 15.Tongwei, Gansu | 35°12' | 105°14' | 444.2 | 441.6 | CT, FM,  WSM | Winter | 2011-2012 | Fan et al. (2013) |
| 16.Luoyang, Henan | 34°38' | 112°28' | 643.4 | 125.1 | CT, FM,  WSM, MOM | Winter | 2004-2005 | Zhang et al. (2008) |
| 17.Luoyang, Henan | 34°30' | 113° | 600 | 227.6 | CT, FM | Winter | 1999-2003 | Su et al. (2004) |
| 18.Fushan, Shanxi | 36°49' | 110°77' | 481.7 | 185.73 | CT, FM | Winter | 1997-1999 | Liu et al. (2001) |
| 19.Taigu, Shanxi | 37°25' | 112°33' | 462.9 | 206.2 | CT, FM,  MOM | Winter | 2003-2004 | Fan et al. (2005a) |
| 20.Luoyang, Henan | 34°38' | 112°28' | 643 | 264.9 | CT, RFM,  WSM | Winter | 2007-2008 | Lv et al. (2012) |
| 21.Dingxi, Gansu | 35°28' | 104°44' | 420 | 191.5 | CT, FM,  RFM | Winter | 2008-2009 | Zhang et al. (2011) |
| 22.Heyang, Shaanxi | 34°10' | 106°20' | 550 | 264.67 | CT, RFM,  MTMC | Winter | 2007-2010 | Han et al. (2014) |
| 23.Tianshui, Gansu | 34°34' | 105°43' | 510 | 222.03 | CT, FM | Winter | 2009-2012 | Lu et al. (2013) |
| 24.Heyang, Shaanxi | 35°14' | 110°09' | 550 | 256.5 | CT, RFM,  MOM | Winter | 2008-2010 | Yang et al. (2011) |
| 25.Taiyuan, Shanxi | 37°47' | 112°35' | 456 | 83.7 | CT, FM,  RFM | Winter | 1996-1997 | Huang et al. (1999) |
| 26.Heyang, Shaanxi | 35°19' | 110°04' | 582 | — | CT, WSM | Winter | 2001-2004 | Zhang et al. (2007) |
| 27.Pingliang, Gansu | 35°16' | 107°30' | 540 | 216 | CT, WSM | Winter | 1979-2002 | Fan et al. (2005b) |
| 28.Linfen, Shanxi | 35°05' | 111°31' | 555 | — | CT, MOM | Winter | 1993-2000 | He et al. (2007) |
| 29.Heyang, Shaanxi | 35°19' | 110°04' | 582 | — | CT, WSM | Winter | 1981-2000 | Zhang et al. (2009) |
| 30.Changwu, Shaanxi | 35°12' | 107°45' | 584.1 | 155.2 | CT, FM | Winter | 2008-2009 | Chen et al. (2010) |
| 31.Dingxi, Gansu | 35°33' | 104°35' | 420 | 75.1 | CT, FM,  RFM | Spring | 2009 | Zhang et al. (2011) |
| 32.Dingxi, Gansu | 35°36' | 104°35' | 420 | 202.6 | CT, FM,  RFM | Spring | 2010 | Zhang et al. (2012b) |
| 33.Dingxi, Gansu | 35°36' | 104°35' | 415 | 183.8 | CT, MOM | Spring | 2010-2011 | Wang et al. (2013) |
| 34.Dingxi, Gansu | 35°37' | 104°36' | 420 | 153.25 | CT, WSM | Spring | 2000-2002 | Jin et al. (2005) |
| 35.Dingxi, Gansu | 35°38' | 104°37' | 360 | 224.4 | CT, FM,  RFM | Spring | 2010-2011 | Zhang et al. (2012c) |
| 36.Dingxi, Gansu | 35°39' | 104°38' | 415 | 152.8 | CT, FM | Spring | 2002 | Du et al. (2004) |
| 37.Dingxi, Gansu | 35°33' | 104°35' | 426.6 | — | CT, WSM | Spring | 1997-1998 | Huang et al. (2005) |
| 38.Dingxi, Gansu | 35°57' | 104°59' | 420 | 195 | CT, FM | Spring | 1999-2000 | Li et al. (2004) |
