# Supplementary material for: Yield Responses of Wheat to Mulching Practices in Dryland Farming on the Loess Plateau
Source: PLoS One. 2015 May 28;10(5):e0127402. doi: 10.1371/journal.pone.0127402 (PMC4447293; doi:10.1371/journal.pone.0127402)
Supplement: S3 Table — (DOCX) [file pone.0127402.s003.docx]

**S3 Table.** Full references of 38 studies included in the meta-analysis.

1. Zhang P, Zhang XF, Wei T, Jia ZK, Ren XL, Ding RX. Effects of furrow planting with ridge film mulching and side planting with flat film mulching on photosynthesis and yield of winter wheat. Agricultural Research in the Arid Areas. 2012a; 30: 32-37 (in Chinese).

2. Liu T, Jia ZK, Zhang R, Zheng JC, Ren SC, Yang BP, et al. Effect of straw mulching on soil moisture and water use efficiency of winter wheat in dryland. Journal of Northwest A&F University (Nat. Sci. Ed.) 2010; 38: 68-76 (in Chinese).

3. Zhang SL, Lars L, Tong YA. Effects of different field management practices on winter wheat yield and water utilization efficiency in Weibei Loess Plateau. Transactions of the CSAE. 2005; 21: 20-24 (in Chinese).

4. Li R, Cui RM, Jia ZK, Han QF, Lu WT, Hou XQ. Effects of different furrow-ridge mulching ways on soil moisture and water use efficiency of winter wheat. Scientia Agricultura Sinica. 2011; 44: 3312-3322 (in Chinese).

5. Wang CR, Tian XH, Li SX. Effects of plastic sheet-mulching on ridge for rainwater-harvesting cultivation on WUE and yield of winter wheat. Scientia Agricultura Sinica. 2004; 37: 208-214 (in Chinese) .

6. Wu JZ, Huang M, Li YJ, Chen MC, Yao YQ, Guo DY, et al. Effects of different tillage systems on the photosynthesis functions, grain yield and WUE in winter wheat. Agricultural Research in the Arid Areas. 2008; 26: 17-21 (in Chinese).

7. Dang TH, Guo D, Qi LH. Effects of wheat yield and water use under dual-mulching mode of plastic film and straw in the dryland farming. Transactions of the CSAE. 2008; 24: 20-24 (in Chinese).

8. Bai LT, Hai JB, Han QF, Jia ZK. Effects of mulching with different kinds of plastic film on growth and water use efficiency of winter wheat in Weibei Highland. Agricultural Research in the Arid Areas. 2010; 28: 135-139 (in Chinese).

9. Liu YH, Jia ZK, Zhang Rui, Liu T, Ma XL. Effects of dual mulching with plastic film and other mulching materials on soil water and WUE in semiarid region. Agricultural Research in the Arid Areas. 2010; 28: 152-157 (in Chinese).

10. Ma XL, Jia ZK, Xiao ES, Wang WY, Liu T, Liu YH, et al. Effects of wheat-residue application on soil water and water use efficiency in the Weibei Loess Plateau. Agricultural Research in the Arid Areas. 2010; 28: 59-64 (in Chinese).

11. Wen PP, Sun M, Deng LF, Zhao WF, Gao ZQ. Effect of deep-plow and mulching during fallow period on soil water and wheat water use efficiency in dryland. Chinese Journal of Eco-Agriculture. 2013; 21: 1358-1364 (in Chinese).

12. Yang XM, Hao MD, Li L, Dong XB. Effects of mulching modes on nutrient uptake and water use of winter wheat in dryland of loess region. Journal of Triticeae Crops. 2013; 33: 1001-1005 (in Chinese).

13. Liao YC, Wen XX, Han SM, Jia ZK. Effect of mulching of water conservation for dryland winter wheat in the loess tableland. Scientia Agricultura Sinica. 2003; 36: 548-552 (in Chinese).

14. Fang RY, Zhao HQ, Fang J. Water saving benefits of different mulching cultivation mode for winter wheat in Weibei High land. Transactions of the CSAE. 2006; 22: 46-49 (in Chinese).

15. Fan YD, Chai SX, Cheng HB, Chen YZ, Yang CG, Huang CX. Effects of mulching on soil moisture in dryland winter wheat field, Northwest China. Chinese Journal of Applied Ecology. 2013; 24: 3137-3144 (in Chinese).

16. Zhang J, Lv JJ, Wang YH, Li JH, Ding ZQ, Yao YQ. Effects of different mulching methods on winter wheat the growth on West of Henan dry-land. Agricultural Research in the Arid Areas 2008; 26: 94-97 (in Chinese).

17. Su ZY, Yang ZL, Wang DL, Cai DX, Yao YQ, Lv JJ, et al. Effect of conservation tillage on water conserving on loess slope-land in west Henan Province. Agricultural Research in the Arid Areas. 2004; 22: 6-8 (in Chinese).

18. Liu LL, Zhang SL (2001) Effects of film covering on yield of winter wheat under the condition of permeable irrigation in the arid region. Agricultural Research in the Arid Areas 19: 48-53 (in Chinese).

19. Fan JH, Song XL, Hao JP. Studies on two effects of water-permeability plastic membrane mulching in rainfed wheat field. Journal of Liaoning Agricultural College. 2005a; 7: 1-5 (in Chinese).

20. Lv JJ, Li JH, Tan ZS, Ding ZQ, Zhang J, Wu JF, et al. Study on wheat and corn yields, soil carbon and water under ridge culture with straw mulch. Journal of Henan Agricultural Sciences. 2012; 41: 68-72 (in Chinese).

21. Zhang SF, Chai SX, Lin YC, Chang Lei, Lu XH. Effects of plastic film mulching on soil moisture in winter wheat field. Journal of Gansu Agricultural University. 2011; 46: 45-52 (in Chinese).

22. Han J, Liao YC, Jia ZK, Han QF, Ding RX. Effects of ridging with mulching on yield and water use efficiency in winter wheat in semi-humid drought-prone region in China. Acta Agronomica Sinica. 2014; 40: 101-109 (in Chinese).

23. Lu QL, Chai SX, Zhang LJ, Yang FR, Zhou J. Water-saving Effect of the full plastic film mulching with soil covered on plastic film on first- crop and second- crop wheat. Journal of Nuclear Agricultural Sciences. 2013; 27: 1903-1911 (in Chinese).

24. Yang HD, Hai JB, Jia ZK, Han QF, Zhang BJ, Ren SC. Effect of different plastic-film mulching in the whole growth period on soil moisture and water use efficiency of winter wheat. Agricultural Research in the Arid Areas. 2011; 29: 27-34 (in Chinese).

25. Huang MJ, Jin FS, Chi BL, Chen QE. A study on characteristics of water consumption in winter wheat under plastic covering. Agricultural Research in the Arid Areas. 1999; 17: 20-23 (in Chinese).

26. Zhang SL, [Lovdahl L](http://apps.webofknowledge.com/OneClickSearch.do?product=WOS&search_mode=OneClickSearch&excludeEventConfig=ExcludeIfFromFullRecPage&colName=WOS&SID=1FYAHruJKj87lgONHqW&field=AU&value=Lovdahl,%20L&cacheurlFromRightClick=no), [Grip H](http://apps.webofknowledge.com/OneClickSearch.do?product=WOS&search_mode=OneClickSearch&excludeEventConfig=ExcludeIfFromFullRecPage&colName=WOS&SID=1FYAHruJKj87lgONHqW&field=AU&value=Grip,%20H&cacheurlFromRightClick=no), [Jansson, PE](http://apps.webofknowledge.com/DaisyOneClickSearch.do?product=WOS&search_mode=DaisyOneClickSearch&colName=WOS&SID=1FYAHruJKj87lgONHqW&author_name=Jansson,%20PE&dais_id=16177431&excludeEventConfig=ExcludeIfFromFullRecPage&cacheurlFromRightClick=no), [Tong YN](http://apps.webofknowledge.com/OneClickSearch.do?product=WOS&search_mode=OneClickSearch&excludeEventConfig=ExcludeIfFromFullRecPage&colName=WOS&SID=1FYAHruJKj87lgONHqW&field=AU&value=Tong,%20YN&cacheurlFromRightClick=no). Modelling the effects of mulching and fallow cropping on water balance in the Chinese Loess Plateau. Soil & Tillage Research 2007; 93: 283-298.

27. Fan TL, Wang SY, Tang XM, Luo JJ, Stewart BA, Gao YF. Grain yield and water use in a long-term fertilization trial in Northwest China. Agricultural Water Management. 2005b; 76: 36-52.

28. He J, Li HW, Wang XY, McHugh AD, Li WY, Gao HW, et al. The adoption of annual subsoiling as conservation tillage in dryland maize and wheat cultivation in northern China. Soil & Tillage Research. 2007; 94: 493-502.

29. Zhang SL, [Lovdahl L](http://apps.webofknowledge.com/OneClickSearch.do?product=WOS&search_mode=OneClickSearch&excludeEventConfig=ExcludeIfFromFullRecPage&colName=WOS&SID=1FYAHruJKj87lgONHqW&field=AU&value=Lovdahl,%20L&cacheurlFromRightClick=no), Grip H, Yong YN, Yang XY, Wang QJ. Effects of mulching and catch cropping on soil temperature, soil moisture and wheat yield on the Loess Plateau of China. Soil & Tillage Research. 2009; 102: 78–86.

30. Chen YH, Zhang SQ, Tian HY, Chen W. Effects of plastic mulch and manure on soil temperature and water consumption of winter wheat. Bulletin of Soil and Water Conservation. 2010; 30: 59-63 (in Chinese).

31. Zhang SF, Chai SX, Lin YC, Chang Lei, Yang CG. Effects of plastic film mulching patterns on soil moisture and spring wheat yields in drought years. Chinese Journal of Agrometeorology. 2011; 32: 368-374 (in Chinese).

32. Zhang PL, Guo TW, Hou HZ, Lu JF. Effects of different cultivation and balanced fertilization on spring wheat yield and water use efficiency in semiarid areas. Agricultural Research in the Arid Areas. 2012b; 30: 132-137.

33. Wang HL, Song SY, Zhang XC, Gao SM, Yu XF, Ma YF. Effects of using plastic film as mulch combined with bunch planting on soil temperature, moisture and yield of spring wheat in a semi- arid area in drylands of Gansu, China. Acta Ecologica Sinica. 2013; 33: 5580-5588 (in Chinese).

34. Jin XJ, Huang GB. Effects of different tillage methods on soil water and water use efficiency in semi-arid area of Gansu. Journal of Soil and Water Conservation. 2005; 19: 109-112 (in Chinese).

35. Zhang PL, Guo TW, Zhang XC, Lv JF, Hou HZ. Effects of different bunch-planting cultivation on spring wheat yield and water use efficiency in the semi-arid area. Acta Agriculturae Boreali-occidentalis Sinica. 2012c; 21: 39-42 (in Chinese) .

36. Du YJ, Li ZZ, Li FM. Effects of plastic fi lm mulch and pre- sowing soil water on growth and yield of spring wheat in semi-arid areas of Loess Plateau. Acta Bot. Boreal. -Occident. Sin. 2004; 24: 404-411 (in Chinese).

37. Huang YL, Chen LD, Fu BJ, Huang ZL, Gong J. The wheat yields and water-use efﬁciency in the Loess Plateau: straw mulch and irrigation effects. Agricultural Water Management. 2005; 72: 209-222.

38. Li FM, Wang J, Xu JZ, Xu HL. Productivity and soil response to plastic ﬁlm mulching durations for spring wheat on entisols in the semiarid Loess Plateau of China. Soil & Tillage Research. 2004; 78: 9-20.
